# Recurrent Kawasaki disease with polyserositis and pulmonary lesions in a 5-year-old boy: a case report

**DOI:** 10.3389/fped.2025.1577361

**Published:** 2025-06-12

**Authors:** Jiahui Weng, Yang Wen, Yiyuan Li

**Affiliations:** ^1^ Department of Infectious Diseases, West China Second University Hospital, and Key Laboratory of Obstetric and Gynecologic and Pediatric Diseases and Birth Defects of the Ministry of Education, Sichuan University, Sichuan, Chengdu, China; ^2^ Department of Orthopedic Surgery, the Second Affiliated Hospital, Zhejiang University School of Medicine, Hangzhou, Zhejiang, China; ^3^ Orthopedics Research Institute of Zhejiang University, Hangzhou, Zhejiang, China

**Keywords:** Kawasaki disease, pulmonary lesions, polyserositis, pediatric, recurrent

## Abstract

**Background:**

Kawasaki disease (KD) is associated with coronary artery alterations, and recurrence of the disease is relatively rare. We here report a case of recurrent KD complicated by polyserositis and pulmonary lesions in a young male patient.

**Case presentation:**

A 5 year old male experienced a recurrence of KD complicated by polyserositis and pulmonary lesions, with concurrent *Shigella bogdii* infection. The simultaneous occurrence of these complications is exceptionally uncommon. An early diagnosis was established, and systemic immunosuppressive therapy was promptly initiated. The therapeutic intervention proved to be effective, leading to a complete resolution of the condition.

**Conclusion:**

KD may induce polyserositis, heart failure, and pulmonary lesions, making early diagnosis and immediate treatment crucial for prognostic enhancement.

## 1 Introduction

Kawasaki disease (KD) is a systemic vasculitis that predominantly affects infants and young children, and has become the leading cause of acquired heart disease in this population. The potentially life-threatening cardiac complications of KD involve transient pancarditis and coronary artery aneurysms, which may lead to ischemic heart disease and myocardial infarction (1, 2). Recurrence of KD is rare, with an incidence of approximately 3%, and is associated with an increased risk of coronary artery aneurysm formation (2). The etiology and pathogenesis of KD remain incompletely understood. Current evidence suggests that KD may involve a multifactorial interplay of regional and ethnic disparities, dietary and environmental factors, dysregulated immunoinflammatory responses, and genetic predisposition. While multiple infectious factors have been associated with KD, no definitive causative pathogen has been identified (3, 4).

Polyserositis, characterized by inflammatory effusions affecting multiple serous membranes, is frequently associated with malignant neoplasms, infectious processes, and autoimmune diseases. The clinical presentation exhibits considerable heterogeneity, with diagnostic confirmation predominantly dependent on advanced imaging techniques. Although serous effusions in the context of systemic vasculitis are most commonly observed in systemic lupus erythematosus, their occurrence in KD is exceptionally rare (5). Pulmonary manifestations, representing an uncommon feature of KD, have been reported in approximately 1.8% of cases, with potential presentations including pleural effusions, pulmonary nodules, or hydropneumothorax (6). The pathophysiological mechanism is hypothesized to involve vasculitis-induced enhancement of vascular permeability, although such clinical manifestations remain infrequent in medium-sized vessel vasculitides.

We herein report a case of recurrent KD complicated by polyserositis and pulmonary lesions in a young male patient, potentially associated with bacterial exposure. The patient achieved complete recovery following treatment with immunoglobulin (IVIG), prednisone, clopidogrel, and antibiotics.

## 2 Case presentation

A 5-year-old male presented to a local hospital with a 1-day history of bilateral cervical lymphadenopathy, with palpable masses approximately 2 cm × 1 cm in size. On the second day, the patient developed a high-grade fever (39.2°C) in the absence of cough, vomiting, or diarrhea. Complete blood count (CBC) revealed marked leukocytosis with a white blood cell (WBC) count of 26.3 × 10^9^/L and neutrophilic predominance (87.3%). Hemoglobin was 125 g/L and platelet count was 195 × 10^9^/L, both within normal limits. C-reactive protein (CRP) was significantly elevated at 128 mg/L. Bacterial lymphadenitis was initially considered. Despite 3 days of intravenous amoxicillin-clavulanate (90 mg/kg/day in three divided doses), followed by 1 day of intravenous oxacillin (100 mg/kg/day in four divided doses), the patient remained febrile and developed progressive cervical lymphadenopathy, complicated by pharyngitis, bilateral non-purulent conjunctival injection, and mild abdominal discomfort, leading to referral to our institution on the 7th day.

Three years ago, the patient was diagnosed with KD based on persistent fever, cutaneous rash, bilateral non-purulent conjunctival injection, and cervical lymphadenopathy. Initial echocardiography showed moderate tricuspid regurgitation and mild mitral regurgitation. The patient responded well to IVIG (2 g/kg) and aspirin therapy. Follow-up echocardiographic evaluations demonstrated complete resolution of cardiac abnormalities without sequelae. One week preceding symptom onset, the patient developed diarrhea following ingestion of barbecued meat and inadequately cooked vegetables. The parents reported no history of SARS-CoV-2 infection in the pediatric patient within the past 3 months.

Physical examination revealed bilateral conjunctival injection, a strawberry tongue, and sclerodactyly of both hands. The tonsils were enlarged with purulent exudate. A palpable cervical mass measuring 5 cm × 5 cm was noted inferior to the left ear, accompanied by overlying erythema and tenderness. The abdomen was soft, with periumbilical tenderness. The liver was palpable 6.5 cm below the right costal margin and approximately 7 cm below the xiphoid process. The remainder of the physical examination was unremarkable.

The laboratory findings revealed marked leukocytosis (WBC 17.3 × 10^9^/L) with a neutrophilic predominance (83.9% neutrophils), accompanied by relative lymphopenia (15% lymphocytes). Additionally, the patient presented with mild anemia (hemoglobin 109 g/L) and reactive thrombocytosis (platelet count 284 × 10^9^/L). Markedly elevated inflammatory markers were observed, including a C-reactive protein (CRP) concentration of 92.4 mg/L and an erythrocyte sedimentation rate (ESR) of 69 mm/h (reference range: 0–20 mm/h). The levels of ferritin, and liver function tests, including aspartate aminotransferase (AST), alanine aminotransferase (ALT), and gamma-glutamyl transferase (γ-GTP), as well as serum sodium and lipid profile, were all within normal limits. However, hypoalbuminemia was evident, characterized by a reduced serum albumin concentration of 32.6 g/L and a decreased prealbumin level of 28 mg/L. Cardiac involvement was suggested by an elevated cardiac troponin I level of 0.267 μg/L (reference range: 0–0.06 μg/L). Stool routine analyses were normal. Screening for tuberculosis, including the purified protein derivative skin test, Interferon-Gamma Release Assay, and blood cultures, yielded negative results. Serial SARS-CoV-2 nucleic acid tests during hospitalization were consistently negative.

**Figure 1:**
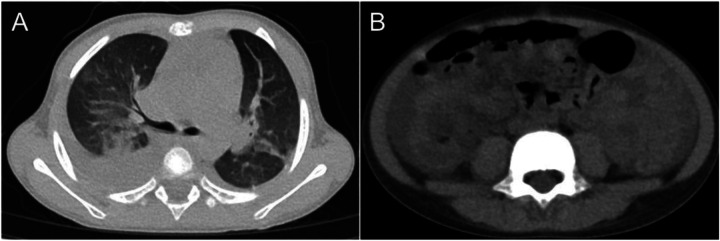
CT imaging of the chest and abdomen: **(A)** nodules and interstitial pneumonia observed in both lungs. **(B)** shadows in the perirectal region and abdominal-pelvic fat space, along with peritoneal thickening.

The patient was initially diagnosed with tonsillitis, with a clinical suspicion of sepsis and/or recurrent, severe KD. Empirical antibiotic therapy with amoxicillin-clavulanate was initiated. On the 9th day, the patient developed abdominal pain, nausea, and vomiting, with periumbilical tenderness. The respiratory rate increased to 50 breaths per minute, oxygen saturation declined to 93%–95%, and the patient exhibited suprasternal and intercostal retractions, with auscultation revealing rales in both lungs. Transthoracic echocardiography revealed cardiomegaly, severe tricuspid regurgitation, moderate mitral regurgitation, and a left ventricular ejection fraction of 58%. Chest and abdominal computer telegram (CT) scans demonstrated gallbladder enlargement with wall thickening and heterogeneous density, blurred fat planes in the rectal and abdominopelvic regions, abdominopelvic effusion, bilateral pulmonary infiltrates and pleural effusions, and a small pericardial effusion (Figure 1).

A diagnosis of severe KD complicated by polyserositis and heart failure was established. On day nine of illness, high-dose IVIG (2 g/kg) was administered, along with clopidogrel for antiplatelet therapy, low-flow oxygen via nasal cannula at 0.5 L/min, ceftriaxone instead of amoxicillin-clavulanate for antimicrobial therapy, and diuretics for the management of serous effusions. By day ten, the patient exhibited clinical improvement, including resolution of fever, reduced conjunctival injection, regression of peripheral edema, improvement of strawberry tongue, and a decrease in the size of cervical lymphadenopathy to approximately 4 cm × 4 cm. Oral prednisone (2 mg/kg/day in two divided doses) was added for anti-inflammatory treatment. The patient remained afebrile thereafter.

Concurrently, stool culture yielded *Shigella boydii*, resistant to ceftriaxone but sensitive to cefoperazone-sulbactam. Accordingly, antibiotic therapy was adjusted to cefoperazone-sulbactam, resulting in resolution of abdominal pain and vomiting. The patient was able to maintain oxygen saturation >95% without supplemental oxygen, and hepatomegaly gradually regressed. Repeat echocardiography 5 days after IVIG administration showed no significant abnormalities. After 10 days of cefoperazone-sulbactam treatment, follow-up chest CT demonstrated resolution of pleural and pericardial effusions and significant absorption of pulmonary infiltrates. Abdominal ultrasonography indicated complete absorption of the peritoneal fluid.

Follow-up echocardiography at 6 months, 12 months, and 2 years post-discharge revealed no abnormalities.

## 3 Discussion

KD is a vasculitis of unknown origin of small and medium caliber blood vessels, and presents with a constellation of clinical features including persistent high fever, bilateral non-purulent conjunctivitis, polymorphous exanthema, cervical lymphadenopathy, and a spectrum of cardiovascular complications. Identifying susceptibility to KD and recognizing typical symptoms may facilitate early diagnosis and improve prognosis (7).

Polyserositis and cardiac failure represent rare complications of KD. A case of KD presenting with severe cardiac tamponade was successfully managed with pulse methylprednisolone therapy (30 mg/kg once daily for 3 days) (8). Current literature confirms that heart failure constitutes an atypical clinical manifestation of KD in pediatric populations (9). Polyserositis can manifest as an early symptom, and delayed diagnosis of KD may lead to severe outcomes, including fatality (10). The exact mechanism underlying polyserositis remains unclear. Heart failure, as a severe complication, may result in pleuroperitoneal effusion. Low sodium levels have been suggested to correlate with anasarca in a child with KD and rotavirus infection (11). Hypoalbuminemia may exacerbate the immune response and is possibly associated with impaired renal tubular sodium reabsorption (12, 13).

In this case, the patient initially presented without respiratory symptoms, but gradually developed dyspnea, pleural effusion, and pulmonary interstitial changes during the course of the illness. Pulmonary involvement is an atypical manifestation of KD, with the majority of pulmonary changes being attributed to interstitial pneumonia, which has the potential to affect multiple organ systems (6, 14). KD complicated by severe pneumonia was recently reported in a patient who recovered well following treatment with intravenous IVIG rather than antibiotics (15). It has been suggested that the pulmonary lesions were caused by capillary exudation, potentially exacerbated by genetic predisposition (16).

Currently, the etiology and pathogenesis of KD remain incompletely understood. Emerging evidence suggests that KD may be associated with diverse pathogens, which initiate T cell-mediated inflammatory and immune cascades, ultimately resulting in vascular endothelial dysfunction and inflammatory vasculopathy (17, 18). Both bacterial and viral infections, as well as genetic susceptibility factors, have been implicated in KD recurrence (19). Notably, patients with recurrent KD demonstrate an elevated risk of developing cardiac complications (20). The patient's case suggests a potential association between enteric bacterial infection caused by *Shigella boydii* and the recurrence of KD.

## 4 Conclusion

KD can precipitate rare yet severe complications, such as cardiac failure, polyserositis, and pneumonia. Early identification and immediate therapeutic management of these complications are critical for optimizing clinical outcomes.

## Data Availability

The datasets presented in this article are not readily available because of ethical and privacy restrictions. Requests to access the datasets should be directed to the corresponding authors.
